# Anti-inflammatory properties of uvaol on DSS-induced colitis and LPS-stimulated macrophages

**DOI:** 10.1186/s13020-020-00322-0

**Published:** 2020-05-07

**Authors:** Shi-yun Du, Hai-feng Huang, Xian-qian Li, Li-xiang Zhai, Qin-chang Zhu, Kai Zheng, Xun Song, Chen-shu Xu, Chen-yang Li, Ying Li, Zhen-dan He, Hai-tao Xiao

**Affiliations:** 1 School of Pharmaceutical Sciences, Guangdong Key Laboratory for Genome Stability & Human Disease Prevention, Shenzhen Key Laboratory of Novel Natural Health Care Products, Innovation Platform for Natural Small Molecule Drugs, Engineering Laboratory of Shenzhen Natural Small Molecule Innovative Drugs, Health Science Center, Shenzhen University, Shenzhen, 518060 China; 2 The Key Laboratory of Pharmacology and Druggability for Natural Medicines, Department of Education, Guizhou Medical University, Guiyang, 550025 Guizhou China; 3 School of Chinese Medicine, Hong Kong Baptist University, Kowloon, Hong Kong, China

**Keywords:** Uvaol, *Apocynum venetum* L., Anti-inflammatory, Macrophage, Colonic inflammation

## Abstract

**Background:**

*Apocynum venetum* leaves are used as a kind of phytomedicine and the main ingredient in some traditional Chinese medicine products for the relief of colitis. To understand the bioactive constituents of *A. venetum* L., we did a phytochemistry study and investigated anti-Inflammatory effects of compounds and explored the underlying mechanisms.

**Methods:**

We isolated compounds from ethanol extract of *A. venetum* L. leaf and detected the most effective compound by NO inhibition assay. We investigated anti-Inflammatory effects on dextran sulfate sodium (DSS)-induced colitis mice and lipopolysaccharide (LPS)-stimulated RAW264.7 cells. The disease activity index was determined by scores of body weight loss, diarrhea and rectal bleeding; histological damage was analyzed by H&E staining; macrophages change in the colon were analyzed by immunohistochemistry (IHC); myeloperoxidase activity was measured by myeloperoxidase assay kits; levels of proinflammatory cytokines were determined by qPCR and ELISA; protein production such as COX-2, iNOS, STAT3 and ERK1/2 were determined by western blotting.

**Results:**

We isolated uvaol from ethanol extract of *A. venetum* L. leaf and found uvaol has excellent potential of inhibiting NO production. We further found uvaol could attenuate disease activity index (DAI), colon shortening, colon injury, and colonic myeloperoxidase activity in DSS-induced colitis mice. Moreover, uvaol significantly reduces mRNA expression and production of pro-inflammatory cytokines (TNF-α, IL-6, IL-1β, and MCP-1) and infiltration of macrophages in colonic tissues of colitis mice. Studies on LPS challenged murine macrophage RAW246.7 cells also revealed that uvaol reduces mRNA expression and production of pro-inflammatory cytokines and mediators. Mechanically, uvaol inhibits the pro-inflammatory ERK/STAT3 axis in both inflamed colonic tissues and macrophages.

**Conclusions:**

*A. venetum* leaf contains uvaol and uvaol has potent anti-inflammatory effects on DSS-induced experimental colitis and LPS-stimulated RAW264.7 macrophage cells. These results suggest uvaol is a prospective anti-inflammatory agent for colonic inflammation.

## Background

Inflammatory bowel disease (IBD) is a chronic inflammatory condition of intestinal mucosa, which includes Crohn’s disease (CD) and ulcerative colitis (UC) [1]. Epidemiological studies show the incidence of IBD has been popular in western countries and it is continuing to rise in developing nations due to the western diet and lifestyle, bringing a substantial burden to patients, health care system and society worldwide [2, 3]. Although the etiology of IBD is not fully understood, genetic determinants, deregulation of the mucosal immune system, dysbiosis of gut microbiota and environmental factors were exhibited to be essential factors in the development of IBD [1]. In clinics, the current treatment for IBD includes 5-aminosalicylic acid (5-ASA), corticosteroids, immunosuppressant agents, biological therapies and antibiotic therapies. Although these existing therapeutic options are effective in treating IBD clinically, they have consequential limitations such as side effects, tolerance of patients, and high medical cost of drugs [4]. Therefore, it is paramount to explore innovative therapies and potential medications for IBD.

The pivotal event of inflammatory response in the colon is the activation of colonic macrophages [5]. As macrophages are majorities of the immune cells, they possess important biological functions in the initiation, maintenance and resolution of inflammation. Activated macrophages by pathogens or inflammatory irritants will induce innate immune response and produce proinflammatory mediators that lead to inflammation [6]. In the inflamed tissue of IBD patients, a significant increase in the number of macrophages was observed, and this increase was also observed in the inflamed colon of acute colitis in different animal models [7–9]. Depletion of macrophages in DSS-induced colitis in mice ceased the development of colitis [10]. Thus, suppression of inflammatory responses in macrophages is considered as a therapeutic intervention for controlling inflammatory responses in IBD.

*Apocynum venetum* L., a plant of *Apocynaceae* family, commonly grows in salt-barren zone in China, is a plant with windbreak and sand fixation ability [11, 12]. *A. venetum* L. is also used as a folk medicine for regulating blood pressure, relax the nervous system, protecting liver and promoting diuresis [13–15]. Although, its biochemical and physiological mechanisms are unclear, *A. venetum* L. has become a sought-after health product for revamping human health in Asian and North American market [13]. To understand the bioactive constituents of *A. venetum* L., we did phytochemistry study and found *A. venetum* leaf contains uvaol, a triterpene compound, which has sublime potential of inhibiting NO production that was verified in this study. Uvaol has been reported to possess various pharmacological properties including anticancer, antimicrobial, and against phosphodiesterase 4D (PDE4D) activities [16–18]. Recently, studies have revealed that uvaol possesses anti-inflammatory effects against ovalbumin-induced pleuritis and eosinophilic inflammation in vivo and phytohemagglutinin (PHA)-stimulated peripheral mononuclear cells (PBMC) in vitro [19, 20]. Moreover, *A. venetum* leaf has been used as a pivotal ingredient in some traditional Chinese medicine products for the relief of colitis [21, 22]. These findings suggest that uvaol is a potential therapeutic compound for inflammatory gastrointestinal diseases yet to be examined. This study aims to investigate anti-inflammatory effects of uvaol on DSS-induced colitis in vivo and LPS-induced RAW264.7 cells in vitro. The manifested therapeutic effects of uvaol on colonic inflammation and inflamed macrophages reveal that it as a potential drug candidate for IBD therapy.

## Methods

### Reagents and drug

*A. venetum* L. was supplied by Altay Gaubau Tea Co Ltd (Shenzhen, China). Uvaol was provided by Baoji Herbest Bio-Tech Co., Ltd (BaoJi, China). Dextran sulfate sodium (DSS) (M.W. = 36000–50000) was purchased from MP Biomedical (Santa Ana, CA, USA). Lipopolysaccharides (LPS) and the BCA protein assay kit were purchased from Beijing Solarbio Science &Technology Co, Lid (Beijing, China). Myeloperoxidase assay kits were obtained from Nanjing Jiancheng Bioengineering institute (Nanjing, China). Mouse interleukin 6 (IL-6), tumor necrosis factor (TNF-α) and IL-1β ELISA kit were supplied by Bio-Swamp Life Science (Wuhan, China). Griess reagent (modified) was purchased from Sigma-Aldrich Corp. (St. Louis, MO, USA). iNOS(13120S), COX-2(4842S), β-actin(4970S), p-ERK (4370T), STAT3(9139T) and p-STAT3 (Tyr705, 9145S) were purchased from Cell Signaling Technology (Beverly, MA, USA). ERK (16443-1-AP) was purchased from Protientech (Wuhan, China).

### Isolation of uvaol from *A. venetum*

**Fig. 1:**
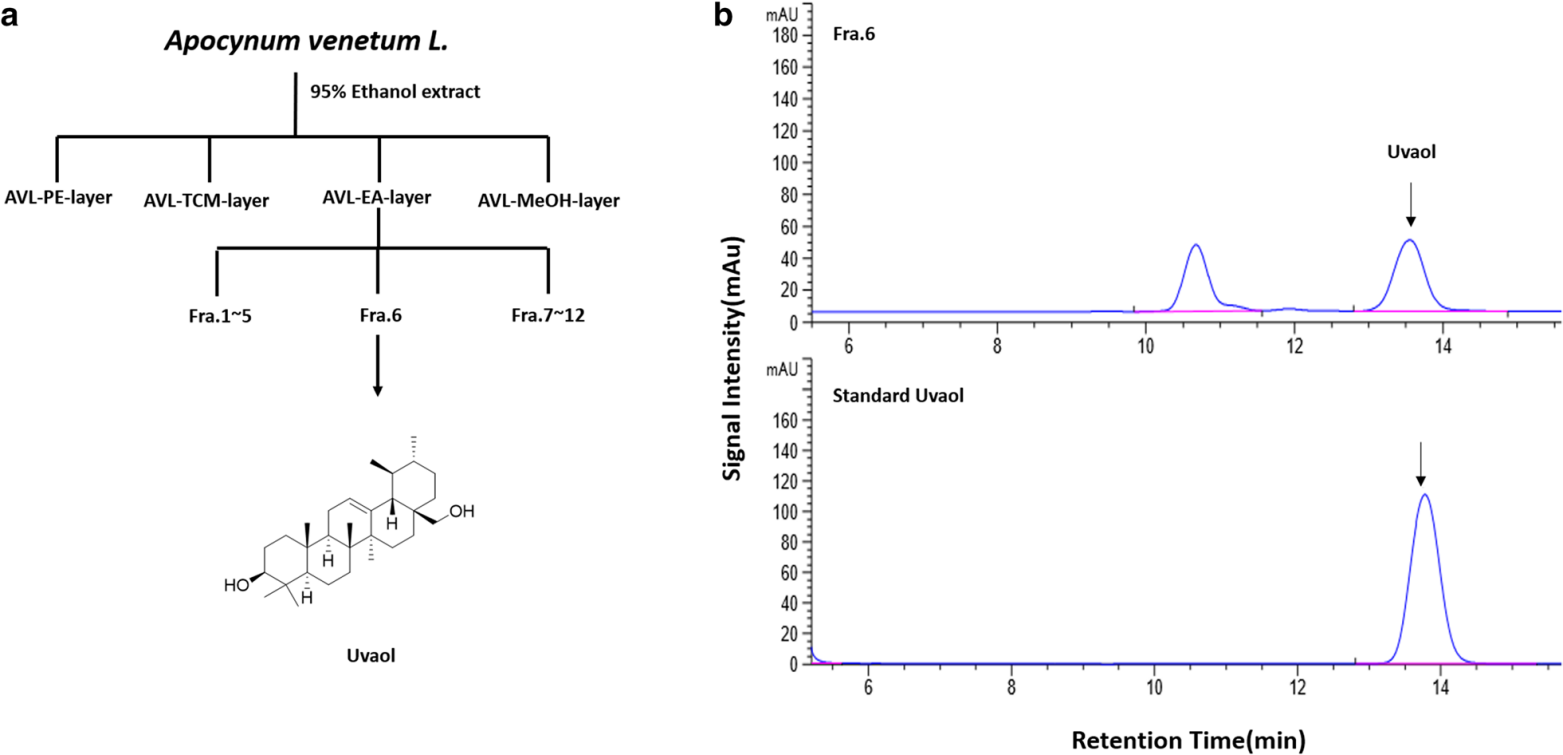
Isolation of uvaol from *A.venetum* L. **a** Isolation flow chart of uvaol. AVL-PE-layer: petroleum ether extract from ethanol extract of *A. venetum* L. leaf, AVL-TCM-layer: chloroform extract from ethanol extract of *A. venetum L.* leaf, AVL-EA-layer: ethyl acetate extract from ethanol extract of *A. venetum L.* leaf, AVL-MeOH-layer: methanol extract from ethanol extract of *A. venetum L.* leaf, **b** chromatograms of a standard uvaol and Fra.6 that contains target compound (210 nm)

The dried leaf of *A. venetum* (10 kg) was extracted 2 times with 95% ethanol at 80 °C and evaporated under reduced pressure (2.5 kg). The part ethanol extract (1 kg) was subjected to silica gel column chromatography (200–300 mesh) and eluted with ethyl acetate to obtain an ethyl acetate extract (25.5 g). The ethyl acetate extract was applied to the silica gel column chromatography, eluted with CH_2_Cl_2_/MeOH (100% CH_2_Cl_2_ → 98% CH_2_Cl_2_ → 96% CH_2_Cl_2_ → 94% CH_2_Cl_2_ → 90% CH_2_Cl_2_ → 100% MeOH) to afford 12 fractions (Fra.1–Fra.12). The fraction 6 (0.5 g) was subjected to octadecyl silica gel column chromatography wit MeOH/H_2_O (30–100%) eluting and further purification by preparative HPLC to obtain the compound (20 mg) [23] (Fig. 1).

Uvaol. Molecular formula: C_30_H_50_O_2_. Amorphous white powder. ^+^TOF–MS m/z: 443[M + H]^+^, 425[M-OH]^+^, ^1^H-NMR(CDCI3, 600 MHz, δ): 5.13(1H, t, *J *= 3.6 Hz, H-12), 3.22(1H, dd, *J *= 11 Hz, 5.0 Hz, H-3), 3.18, 3.53(1H, d, *J *= 11.0 Hz, H-28), 0.93(3H, s, H-29), 0.81 (3H, d, *J *= 5.9 Hz, H-30), 0.79, 0.95, 0.99, 1.00, 1.10 (3H, s, H-23 ~ 27); ^13^C NMR (CDCI3, 151 MHz, δ) 15.63(C-24), 15.71(C-25),16.77(C-30),17.37(C-26), 18.33(C-6), 21.35(C-29), 23.3(C-11), 23.32(C-27), 23.38(C-16),26(C-15), 27.25(C-2), 28.13(C-23), 30.62(C-21), 32.81(C-7), 35.19(C-22), 36.87(C-10), 38.01(C-17), 38.78(C-1), 39.35(C-20), 39.43(C-19), 40(C-8), 42.04(C-14), 47.65(C-9), 54.01(C-18), 55.15(C-5), 69.95(C-28), 79.03(C-3), 125.04(C-12), 138.6(C-13).

### Animals and experiment

Male C57BL/6 mice aged 6–8 weeks (weighed 18–22 g) were purchased from Beijing Vital River Laboratory Animal Technology Co., Ltd. (Beijing, China) and housed in temperature 25 ± 2 °C environment with a 12 h light/dark cycle. The mice were fed with a standard rodent diet with free access to drinking water to adapt to the new environment for 1 week. Control group was given a normal drinking water and diet, the other mice were given 1.8% (v/w) DSS in drinking water for 1 week, which made the mice suffer from acute colitis. Subsequently, they were randomly divided into four groups: DSS model group, CsA group (25 mg/kg CsA, *i.p.*), two uvaol groups (100 mg/kg and 50 mg/kg, *i.p*.). Weight and disease activity index (DAI) scores are measured every 2 days. DAI was determined as described previously [24]. Briefly, the scores of body weight loss, stool consistency and stool blood were calculated based on standard parameters. Fecal occult blood was detected with the hemoccult sensa slides according to the manufacturer’s protocols (Beckman Coulter, Inc., Brea, CA, USA). After 7 days of treatment, all mice were euthanized, blood and colon were harvested for further experiments.

### Cell culture and viability assay

RAW264.7 cells were purchased from the ATCC and cultured with RPMI-1640 containing 10% fetal bovine serum, 100 Units/ml penicillin and 100 µg/ml streptomycin in an incubator at 37 °C in a humidified atmosphere with 5% CO_2_. Cells at a concentration of 1.5 × 10^4^ cells/well were seeded in a 96-well plate overnight and treated with different concentrations of uvaol (0, 0.0625 μM, 0.125 μM, 0.25 μM, and 0.5 μM) for another 24 h, then cell viability was measured by CCK-8 kit.

### NO inhibition assay

RAW264.7 cells were seeded into 48-well plates at a density of 5 × 10^4^/well overnight and incubated without or with uvaol (0.25 µM or 0.125 µM) for 1 h, stimulated with LPS at a final concentration of 1 µg/ml for 24 h at 37 °C, 5% CO_2_ incubator. Supernatants were collected and measured using the Griess reagent following the manufacturer’s instructions.

### Western blotting analysis

Proteins from RAW264.7 cells and colon tissues were extracted with RIPA buffer (Thermo Scientific, Rockford, IL, USA) containing protease and phosphatase inhibiter. Protein concentrations were quantified by BCA assay kit (Beyotime, Shanghai, China). Samples of proteins were separated by 8% or 6% SDS–PAGE gels (Beyotime, Shanghai, China) and transferred onto polyvinylidene fluoride (PVDF) membranes. After blocked with 3% BSA for 2 h, membranes were incubated with the following primary antibodies for 24 h at 4 °C: β-actin (1:1000), iNOS (1:1000), COX-2 (1:1000), STAT3 (1:1000), pSTAT3 (1:1000), ERK (1:2000) and p-ERK (1:1000). After incubation with HRP-linked anti-rabbit or anti-mouse IgG antibodies (1:2000), protein bands were visualized with Pierce™ ECL western blotting substrate (Thermo Scientific, Rockford, IL, USA).

### ELISA analysis

Colonic tissues were homogenized with lysis buffer to extract total protein and centrifuged at 12000 g at 4 °C for 10 min, and the supernatants were collected for the determination of levels of the cytokines. The concentration of TNF-α, IL-6 and IL-1β in the supernatant of LPS-stimulated RAW264.7 cells and colonic tissues were detected by ELISA kits respectively according to manufacturer’s protocols. The total protein in colon tissues sample was measured by the BCA protein assay kit.

### RNA extraction and qRT-PCR analysis

Total RNA of RAW264.7 and colon tissues were extracted by Trizol reagent (Invitrogen, Carlsbad, CA) and followed by reverse transcriptase (TaKaRa, Kusatsu, Shiga, Japan) for cDNA. Quantitative PCR was performed by using a Real Time PCR system with SYBR Green Master (Roche, Basel, Switzerland). Target-gene transcriptions were normalized to β-actin and data were analyzed by 2^−ΔΔCT^ method. The primer sequences were as follows: β-actin (F:GGCTGTATTCCCCTCCATCG; R: CCAGTTGGTAACAATGCCATGT), TNF-α (F: CAGGCGGTGCCTATGTCTC; R: CGATCACCCCGAAGTTCAGTAG), IL-6: (F: CTGCAAGAGACTTCCATCCAG; R: AGTGGTATAGACAGGTCTGTTGG), IL-1β: (F: GAAATGCCACCTTTTGACAGTG; R: TGGATGCTCTCATCAGGACAG), MCP-1: (F: TTAAAAACCTGGATCGGAACCAA; R: GCATTAGCTTCAGATTTACGGGT).

### H&E staining and histological analysis

For the histological examination, distal colon specimens were fixed in 4% formalin for 24 h and embedded in paraffin, stained with hematoxylin and eosin (H&E), and then subjected to blind analysis and scored as previously described [24].

### Immunohistochemical analysis

Colonic tissues were fixed in 4.0% buffered paraformaldehyde. Sectioned samples were deparaffinized in xylene and a series of graded alcohol. Sections were further microwaved in citrate buffer (pH 6.0) to repair antigen and quenched endogenous peroxidase with 3% hydrogen peroxide, and blocked with 3.0% bovine serum albumin (BSA) and incubated with mouse anti-rabbit F4/80 antibody (GB11027, Servicebio, Hubei, China, 1:1000 dilution) at 4 °C overnight. The sections were washed with PBS (PH 7.4) three times and incubated with HRP-linked goat anti-rabbit IgG antibody (GB23303, Servicebio, Hubei, China, 1:200 dilution). Sections were then washed with PBS, and incubated by DAB substrate kit (G1211, Servicebio, Hubei, China) and counterstained with hematoxylin. After dehydration with a series of increasingly concentrated ethanol, sections were mounted with neutral gum. The number of macrophages in colonic tissues were quantified with integral optical density (IOD) by Image Pro-Plus v.6.0 software.

### Statistical analysis

All data were analyzed with GraphPad PRISM 6.0 (GraphPad Software Inc., San Diego, CA, USA) software using one-way ANOVA followed by Bonferroni’s multiple comparisons test. All quantitative values were expressed as the mean ± SEM. Differences were deemed significant when *P *< 0.05.

## Results

### Uvaol improves clinical symptoms and suppresses colon inflammation of DSS-treated mice

**Fig. 2:**
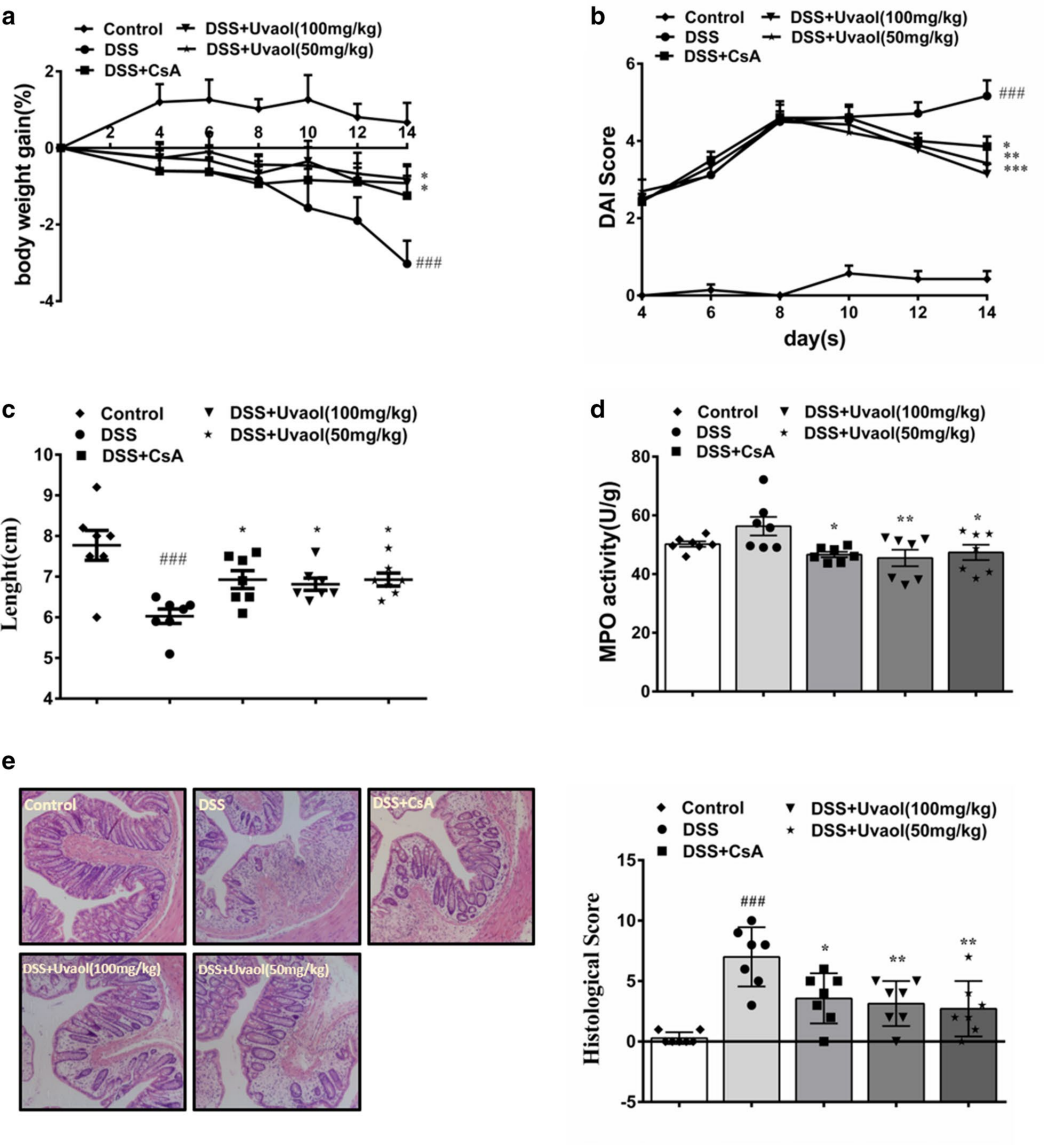
Effects of uvaol on body weight gain (**a**), disease activity index (DAI) (**b**), colon length (**c**), myeloperoxidase (MPO) activity (**d**) and histological damage (magnification of 10 × for representative H&E staining images) (**e**) of DSS-treated mice. Uvaol was administered to mice from day 8 to day 14. Weight and DAI scores are measured every 2 days. DAI was determined by combining scores of (i) body weight loss, (ii) stool consistency, and (iii) stool bleeding. On day 14, the mice were sacrificed, the colon lengths and histological score of colons were measured. Data are expressed as mean ± SEM, n = 7.^###^*P *< 0.001 and^##^*P *< 0.01, compared with control group; ***P < 0.001, **P < 0.01 and *P < 0.05, compared with DSS group

As indicated in Fig. 2a, in comparison with the control group, the body weight gain of DSS-treated mice was lower during the duration of experiment and ANOVA showed significant overall differences between all groups for decreased body weight on day 14 (F = 7.121, *P *= 0.0004). However, this decrease was greatly abrogated by treating with uvaol at dosage of 50 and 100 mg/kg respectively. In parallel, ANOVA showed significant differences between all groups on disease activity index (DAI) score (F = 32.38, *P *< 0.0001). Mice after DSS treatment showed high DAI scores in comparison of normal mice, while both positive agent cyclosporin A (CsA) and uvaol could decrease DAI scores significantly (Fig. 2b). As well, DSS treatment curtail the colon length of mice and this shortening was greatly improved after administration with uvaol and CsA, and ANOVA showed significant overall differences between all groups (F = 7.287, *P *= 0.0003) (Fig. 2c). In mice, the diarrhea and stool blood caused by daily DSS treatment are steered by colonic inflammation and damage. As shown in Fig. 2d, ANOVA showed significant overall differences between all groups for myeloperoxidase (MPO) activity (F = 3.540, *P *= 0.0176). Compared with the control group, the colonic MPO activity of DSS model group was significantly increased, whereas the increase was significantly rescued by both uvaol and CsA administration. As well, severe crypt destruction and inflammatory cell infiltration were observed in the histological sections from DSS-treated mice. Both uvaol and CsA could reduce the histopathological manifestation of colitis (Fig. 2e). The histological scores were also substantially decreased in the uvaol- and CsA-treated groups, and ANOVA showed significant differences between all groups (F = 10.56, *P *< 0.0001). Collectively, these present results reveal that 100 mg/kg and 50 mg/kg dosages of uvaol both exert a potent therapeutic effect against DSS-induced colitis in mice.

### Uvaol reduces macrophage infiltration, mRNA expression and production of pro-inflammatory mediators in colon tissues of DSS-treated mice

**Fig. 3:**
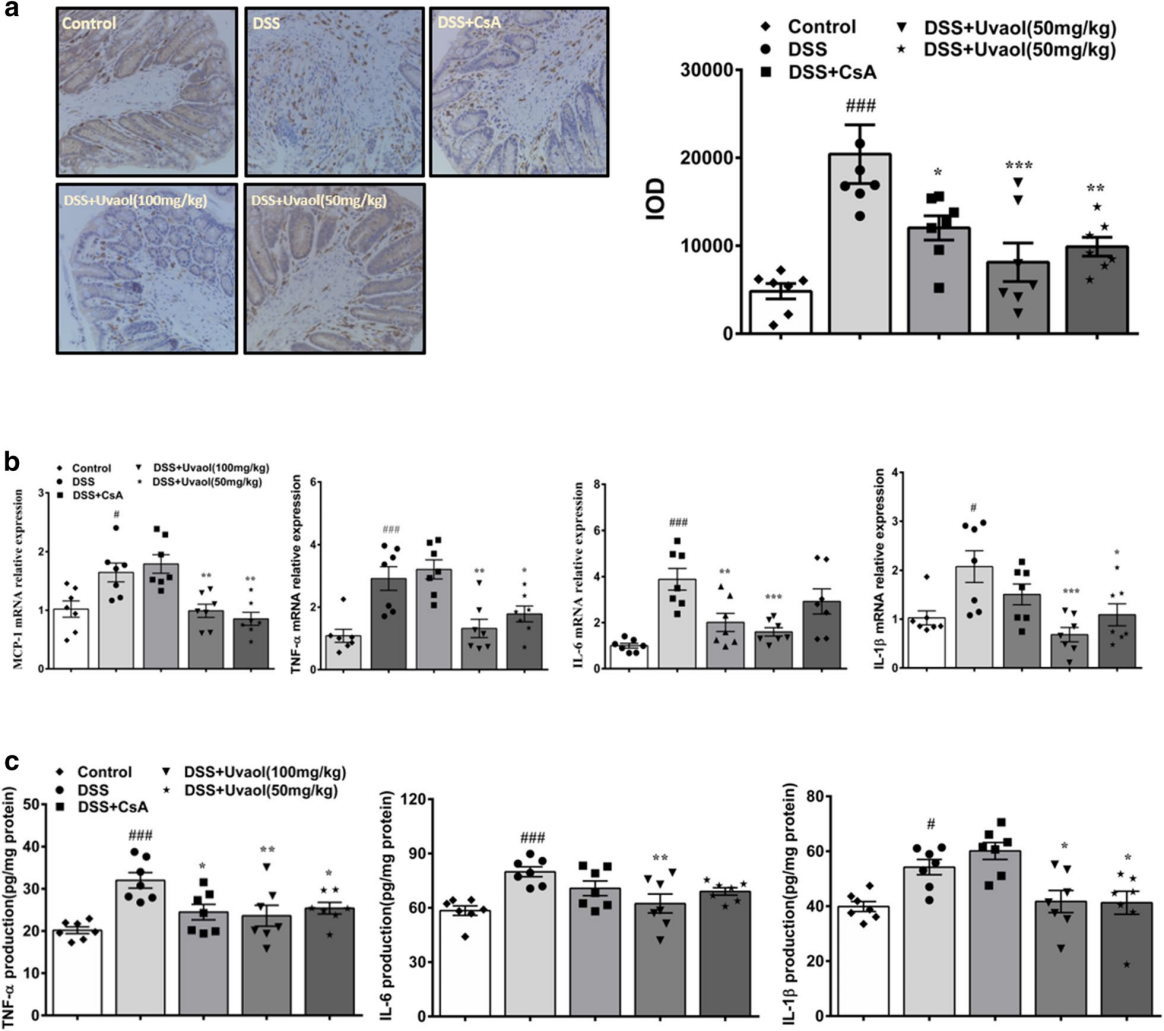
Effects of uvaol on pro-inflammatory macrophage infiltration (**a**), mRNA expression and production of pro-inflammatory mediators (**b**–**c**) in colon tissues of DSS-treated mice. Colitis was induced in all groups except the control group. Uvaol was administered to mice from day 8 to day 14. On day 14, the mice were sacrificed, and mRNA expression and production of pro-inflammatory mediators such as MCP-1, TNF-α, IL-1β and IL-6 in colon tissues were determined by RT-PCR and ELISA. Pro-inflammatory macrophages in colon tissues were stained by F4/80 (magnification of 20 × for representative F4/80 staining images). Integrated optical density(IOD)were analyzed by Image pro-Plus v.6.0. Data are expressed as mean ± SEM, n = 7.^###^*P *< 0.001,^##^*P *< 0.01 and ^#^*P *< 0.05, compared with control group; ***P < 0.001, **P < 0.01 and *P < 0.05, compared with DSS group

As presented in Fig. 3a, ANOVA revealed significant differences between all groups for macrophage infiltration (F = 8.684, *P *< 0.0001). Compared with the control group, DSS treatment resulted in a five-fold increase of macrophages in colon tissues and this accumulation of macrophages were significantly suppressed by intraperitoneal administration of uvaol. For mRNA expression of pro-inflammatory mediators in colon tissues of colitic mice, ANOVA divulged significant differences between all groups for MCP-1 (F = 9.541, *P *< 0.0001), TNF-α (F = 10.61, *P *< 0.0001), IL-1β (F = 5.869, *P *= 0.0013) and IL-6 (F = 9.049, *P *< 0.0001) mRNA expression (Fig. 3b). However, these increases were greatly extricated by uvaol. In parallel, ANOVA also revealed significant differences between all groups for TNF-α (F = 6.062, *P *= 0.0011), IL-1β (F = 7.762, *P *= 0.0002) and IL-6 (F = 5.457, *P *= 0.0020) production in colon tissues of DSS-treated mice. Also, the administration of uvaol significantly decreased protein production of pro-inflammatory cytokines TNF-α, IL-1β and IL-6 in colon tissues of DSS-treated mice (Fig. 3c).

### Uvaol inhibits mRNA expression and production of inflammatory mediator of LPS-stimulated RAW264.7 cells

**Fig. 4:**
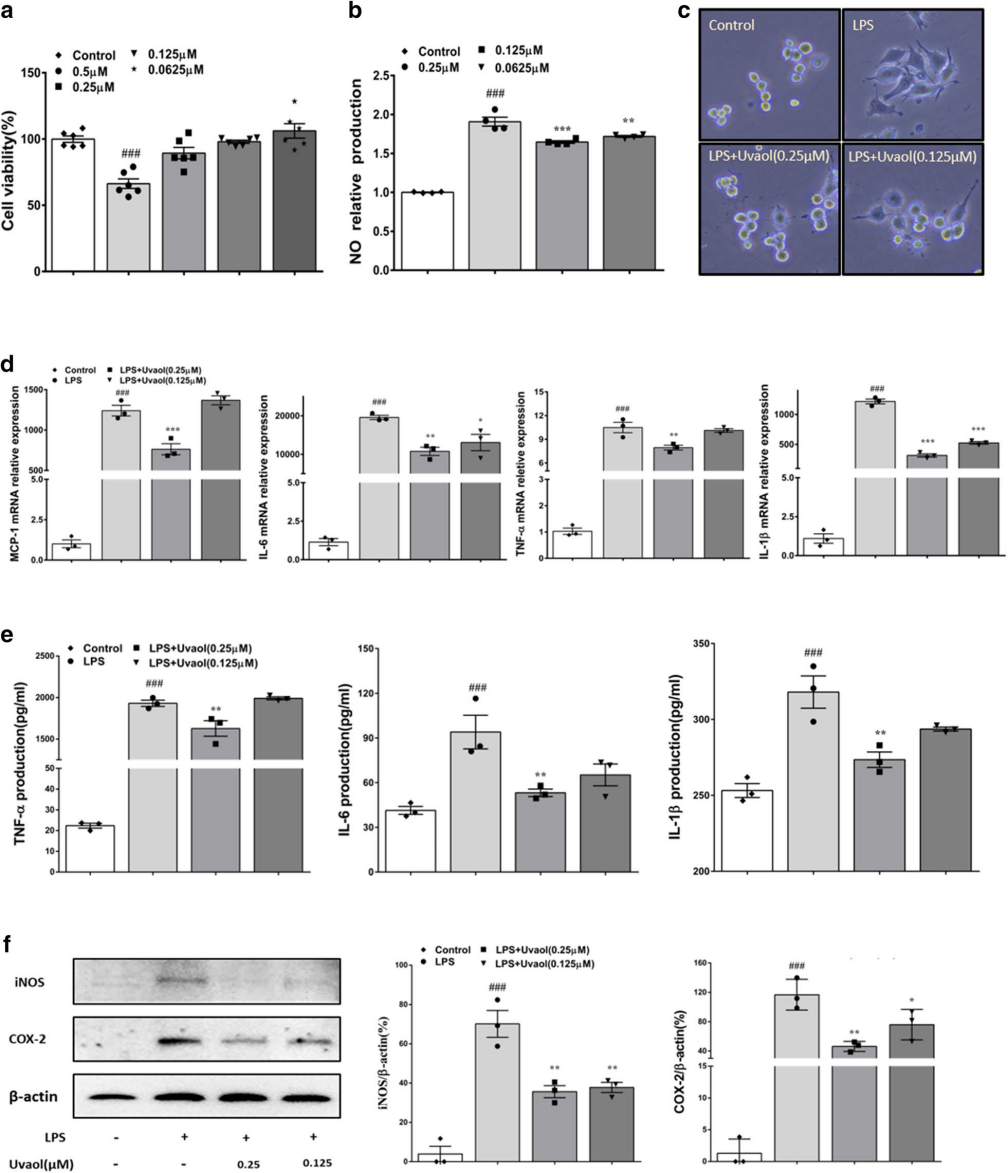
Uvaol inhibits mRNA expression and production of inflammatory mediators of LPS-stimulated RAW264.7 cells. **a** Cytotoxicity of uvaol on RAW264.7 cells. Uvaol was incubated with RAW264.7 cells for 24 h, and cell viability was detected by CCK8 kit. Data are expressed as mean ± SEM, n = 6. **b** NO release in LPS- stimulated RAW264.7 cells. Data are expressed as mean ± SEM, n = 4. **c** The microscopic morphological changes of LPS-induced RAW264.7 cells (magnification 20 ×). **d** mRNA expression of TNF-α, MCP-1, IL-6 and IL-1β of LPS-stimulated RAW264.7 cells. **e** TNF-α, IL-6 and IL-1β protein concentration in supernatants of LPS-stimulated RAW264.7 cells. **f** COX-2 and iNOS expressions of LPS-stimulated RAW264.7 cells. Data are expressed as mean ± SEM, n = 3. The images shown are representatives of three independent experiments.^###^*P *< 0.001 and^##^*P *< 0.01, compared with control group; ***P < 0.001, **P < 0.01 and *P < 0.05, compared with LPS- stimulated group

As exhibited in Fig. 4a, ANOVA illustrated significant differences between all groups for cell viability (F = 17.58, *P *< 0.0001). RAW 264.7 cells treated with uvaol at a concentration range of 0.0625 μM to 0.25 μM showed no significant cytotoxicity as compared with control group. For nitric oxide (NO) production, ANOVA revealed significant differences between all groups (F = 166.4, *P *< 0.0001) (Fig. 4b). RAW264.7 cells were exposed to LPS stimulation, the NO levels were significantly increased in comparison of LPS unstimulation and treatment with different concentrations of uvaol significantly suppressed NO production of LPS-stimulated RAW264.7 cells in a dose-dependent manner. LPS stimulation altered the morphology of RAW264.7 cells. As shown in Fig. 4c, RAW264.7 cells were small, round and bright at normal status and became larger and pseudopods extend wider after LPS stimulation. However, this change in morphology was liberated by uvaol (0.125 and 0.25 μM). Furthermore, LPS stimulation increased mRNA expression and production of pro-inflammatory cytokines. As shown in Fig. 4d, e, ANOVA revealed significant differences between all groups for mRNA expression of TNF-α (F = 128.5, *P *< 0.0001), IL-1β (F = 368.3, *P *< 0.0001), IL-6 (F = 44.83, *P *< 0.0001) and MCP-1 (F = 125.3, *P *< 0.0001); as well as protein production of TNF-α (F = 328.3, *P *< 0.0001), IL-1β (F = 19.06, *P *= 0.0005) and IL-6 (F = 10.46, *P *= 0.0038). Notably, these alterations were significantly relieved by uvaol at a concentration of 0.25 μM, but slightly rescued by uvaol at a concentration of 0.125 μM. In parallel, ANOVA also showed significant differences between all groups for protein expression of iNOS (F = 37.57, *P *< 0.0001) and COX-2 (F = 30.71, *P *< 0.0001). Protein levels of iNOS and COX-2 were significantly upregulated in response to LPS stimulation, and these can be reversed by treatment of uvaol (Fig. 4f).

### Uvaol inhibits STAT3 and ERK activation in vivo and vitro

**Fig. 5:**
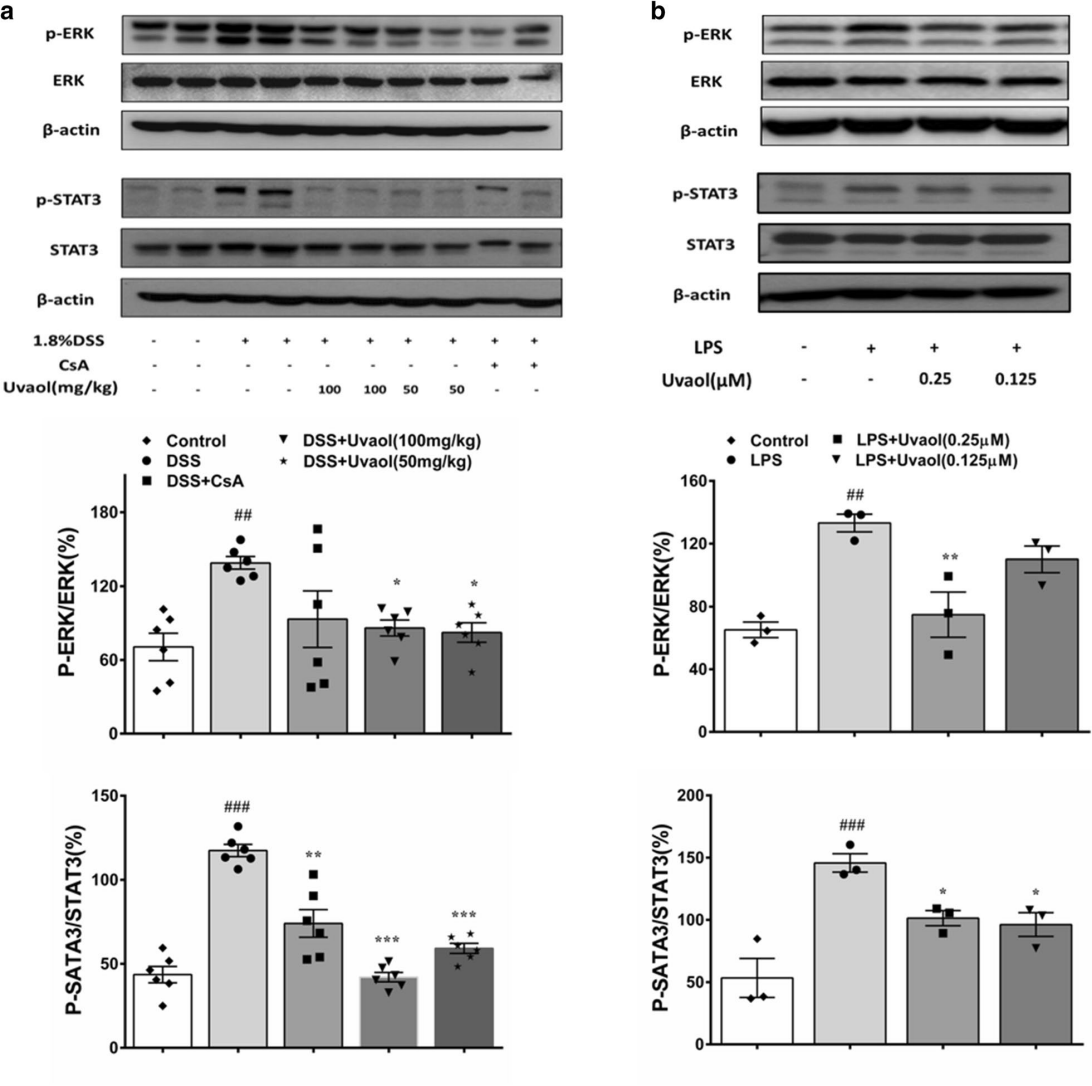
Uvaol inhibits STAT3 and ERK activation in colon tissues of DSS-treated mice and LPS-stimulated RAW264.7 cells. **a** STAT3, p-STAT3, ERK and p-ERK expressions in colon tissues of DSS-treated mice. Colitis was induced in all groups except the control group. Uvaol and CsA were administered to mice from day 8 to day 14. On day 14, the mice were sacrificed, and protein expression of STAT3, p-STAT3, ERK and p-ERK in colon homogenates were determined by western blotting. Data are expressed as mean ± SEM, n = 6. **b** STAT3, p-STAT3, ERK and p-ERK expression in LPS-stimulated RAW264.7 cells. RAW264.7 cells were treated with 1 μg/mL of LPS in the absence or presence of designated concentrations of uvaol for 3 h, Data are expressed as mean ± SEM, n = 3. The images shown are representatives of three independent experiments.^###^*P *< 0.001 and^##^*P *< 0.01, compared with control group; ***P < 0.001, **P < 0.01 and *P < 0.05, compared with DSS or LPS- stimulated group

As presented in Fig. 5a, we detected the effects of uvaol on the expression of STAT3 and p-STAT3 in colon tissues of colitic mice by western blotting, ANOVA revealed significant differences between all groups for p-STAT3 expression (F = 39.63, *P *< 0.0001). After DSS treatment, p-STAT3 expression was significantly increased, while this increase can be reduced by both 100 and 50 mg/kg of uvaol. Further, we next detected the effects of uvaol on ERK and p-ERK expression, and found significant differences between all groups for p-ERK expression (F = 4.412, *P *= 0.0078) by ANOVA. DSS also elevated p-ERK expression, while both 100 and 50 mg/kg uvaol could significantly suppress this elevation (Fig. 5a). Moreover, we also detected the effects of uvaol on STAT3 and ERK activation in LPS-stimulated RAW264.7 cells. As shown in Fig. 5b, ANOVA revealed significant differences between all groups for p-ERK expression (F = 11.79, *P *= 0.0026) and p-STAT3 expression (F = 13.31, *P *= 0.0018), and 0.25 μM uvaol could significantly suppress ERK and STAT3 phosphorylation upon LPS stimulation.

## Discussion

In the current study, we observed that administration of uvaol attenuate the severity of DSS-induced colitis in mice, as affirmed by reduced clinical manifestations, colon shortening, histological damage and colonic myeloperoxidase activity, which is associated with suppression of colonic macrophage activation via inhibition of ERK/STAT3 signaling pathway.

Among the animal models established for studying the pathogenesis of IBD, as well as screening of potential therapeutic interventions, DSS-induced colitis mouse model is one of the most commonly used [25]. This model is elementary and reproducible and its clinical symptoms are similar to ulcerative colitis, such as weight loss, severe diarrhea, rectal bleeding, superficial ulceration, and mucosal damage [24, 25]. In the present study, mice treated with one-week 1.8% DSS drinking water developed typical symptoms of clinical colitis, including body weight loss, stool consistency and bleeding, which indicated that colitic model used in the experiment has been successfully established. Our data from body weight gain, disease activity index score and colon length revealed these typical symptoms of clinical colitis were well emancipated by uvaol administration. Additionally, the histological examination also showed DSS treatment resulted in extensive mucosal inflammation as crypt destruction, lesions, and inflammatory cell infiltration, and our data also clearly showed that uvaol treatment could attenuate crypt destruction and inflammatory cell infiltration of coltic mice with a lower histological score. MPO is a marker of tissue damage and neutrophil infiltration, which is closely linked to the severity of IBD [4]. In the present study, we found that colonic MPO activity in DSS-induced colitis mice was drastically increased as compared with the control mice, whereas the MPO activities in uvaol group were significantly suppressed. Notably, these data indicated that uvaol is a potent therapeutic agent against DSS-induced colitis in mice.

Innate immune system provides the first line of defense against external insults by triggering strong inflammatory response, while causing infiltration of innate immune cells in the intestine of IBD patients. There are a large number of macrophages in the intestine, as an important part of innate immunity and participates in the pathological process of IBD, which play a vital role in maintaining the intestinal homeostasis [26, 27]. Previous studies have verified that a significant increase in the number of macrophages is found in both inflamed colon tissues of IBD patients and colitis animals [7, 9, 27]. In the current study, treatment with DSS resulted in a fivefold increase of macrophages in colon tissues which was consistent with previous report [8], and administration of uvaol could significantly suppress macrophage accumulation. Zigmond E et al. reported that the accumulated macrophages in the inflamed colon were recruited and differentiated from Ly6Chi monocytes in the bloodstream, they are different from resident intestinal macrophages and present as a proinflammatory phenotype [28]. The recruitment of these proinflammatory macrophages was mediated by monocyte chemoattractant protein (MCP-1), which can bind to the chemokine (C–C motif) receptor 2 (CCR2) on circulating monocytes to attract monocytes migrating into inflamed tissues [8]. Accumulated colonic proinflammatory macrophages were activated in the context of colon inflammation, resulting in excessive secretion of proinflammatory cytokines, such as TNF-α, IL-1β and IL-6, which exacerbates colonic inflammation [8, 29]. In the present study, our data showed that uvaol could not only significantly suppress macrophage accumulation, but also diminishes the mRNA levels of MCP-1, TNF-α, IL-1β and IL-6, and the production of TNF-α, IL-1β and IL-6 in the colon tissues of DSS-treated mice in a dose-dependent manner. Our studies in vitro also confirmed that uvaol could reduce LPS challenged mRNA expression and (or) production of pro-inflammatory cytokines (NO, MCP-1, TNF-α, IL-1β and IL-6) and mediators (iNOS and COX-2) in macrophage RAW246.7 cells. These results suggest that uvaol exerts potent anti-inflammatory effects in vivo and in vitro.

It is eminent that the signal transducers and activators of transcription (STAT) pathways play a central role in the regulation of cytokines and the inflammatory response, especially STAT3 [30]. In actively inflamed colons from IBD patients, STAT3 has been found to be activated [31]. Increased STAT3 level in macrophages are classified as the M1 proinflammatory phenotype [32]. As well, STAT3 activation in T-cells directly contributes to colitis [33]. In the present study, we found the phosphorylation of STAT3 was significantly increased in both colon tissues of DSS-induced colitis mice and LPS challenged macrophage RAW246.7 cells, indicating that STAT3 was activated under inflammatory microenvironment. It has been reported that STAT3 protein contains a specific Ser residue in its sequence and it can be recognized and phosphorylated by ERK kinase [34, 35], and our data also found DSS and LPS elevated ERK phosphorylation, suggesting ERK was also activated in flamed colon and LPS challenged macrophages. Notably, administration of uvaol significantly decreased the phosphorylation of STAT3 and ERK in colon tissues of DSS-induced colitis mice and LPS challenged macrophages. Taken together, these data suggest that uvaol exerts anti-inflammatory effects by inhibiting the ERK/STAT3 signaling pathway.

## Conclusions

**Fig. 6:**
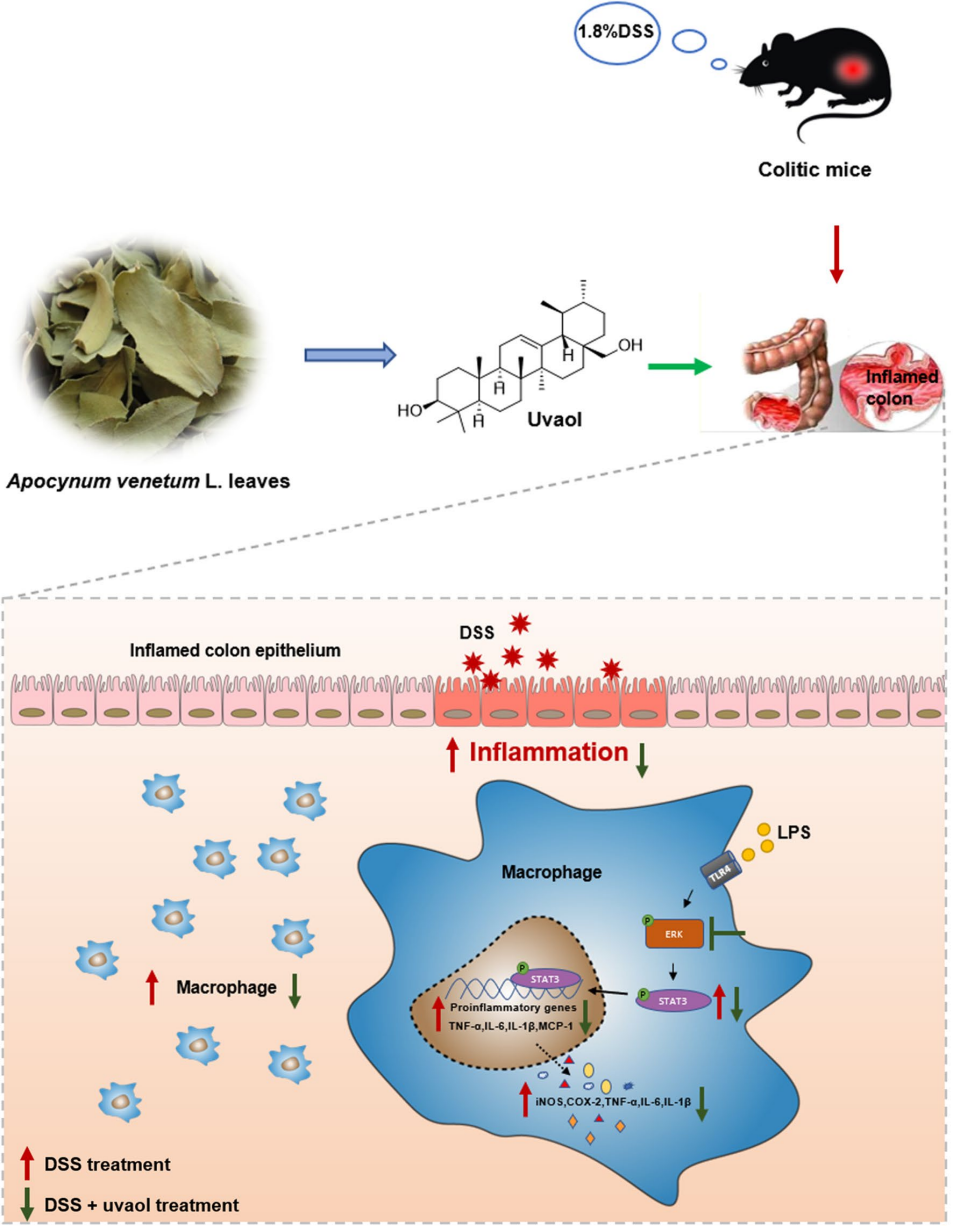
Possible mechanism of uvaol in alleviating DSS-induced experimental colitis via regulation of macrophage pro-inflammatory responses. DSS treatment induced macrophage infiltration and pro-inflammatory responses in the colon. Upon the stimulation by inflammatory stimulus such as TNF-α, LPS in the inflamed colonic circumstance, ERK/STAT3 signal was activated to promote transcription of pro-inflammatory genes TNF-α, IL-1β, IL-6, etc. and then the release of pro-inflammatory cytokines TNF-α, IL-6, IL-1β, COX-2 and iNOS. Uvaol could inhibit ERK and STAT3 activation to suppress the transcription of pro-inflammatory genes and the release of pro-inflammatory cytokines and further improve clinical symptoms and suppress colon inflammation of DSS-treated mice

The present study designates that uvaol is eloquent in alleviating DSS-induced colonic inflammation, which is endorsed by improving the clinical symptoms of colitis, decreasing macrophage infiltration and pro-inflammatory cytokines release in vivo, together with suppressing pro-inflammatory macrophage activation in vitro (Fig. 6). The propitious effect is partially depended on suppression of inflammatory responses of colonic macrophages via inhibition of ERK/STAT3 signaling pathway. Although, bioavailability of uvaol in *A. venetum* L. need further augmented research. Herein, it can be concluded that uvaol is a promising candidate in treatment of IBD.

## Data Availability

The datasets used and/or analyzed during the current study are available from the corresponding author on reasonable request.

## Abbreviations

IBD: Inflammatory bowel disease
CD: Crohn’s disease
UC: Ulcerative colitis
5-SAS: 5-aminosalicylic acid
DSS: Dextran sulfate sodium
LPS: Lipopolysaccharide
COX-2: Cyclooxygenase-2
PDE4D: hosphodiesterase 4D
iNOS: Inducible nitric oxide synthase
NO: Nitric oxide
DAI: Disease activity index
IHC: Immunocytochemistry
MPO: myeloperoxidase
IOD: Integrated optical density
IL: Interleukin
TNF: Tumor necrosis
MCP-1: Monocyte chemotactic protein-1
STAT3: Signal transducer and activator of transcription 3
ERK: Extracellular regulated protein kinases
